# Seasonal Variation in Bait Attractiveness Among Six Mediterranean Pest Ant Species: Implications for Toxic Bait Development

**DOI:** 10.3390/insects17090945

**Published:** 2026-09-10

**Authors:** Sílvia Abril, Àlex López, Mara Moreno-Gómez

**Affiliations:** 1 Departament de Ciències Ambientals, Universitat de Girona, C. Maria Aurèlia Capmany 69, 17003 Girona, Spain; alexlopezcerda@gmail.com; 2 Research and Development (R&D) Insect Control Department, Henkel Ibérica S.A, 08005 Barcelona, Spain; mara.moreno@henkel.com

**Keywords:** ant nutritional ecology, macronutrient preference, seasonal nutritional shifts, toxic bait optimization

## Abstract

Toxic baits are central to professional ant management, but their efficacy depends on matching macronutrient composition to target species’ preferences, which may vary within species and season. Comparative data across co-occurring Mediterranean ant pests are scarce. We quantified protein, carbohydrate, and mixed-diet preferences in six Mediterranean ant pests—*Crematogaster scutellaris*, *Pheidole pallidula*, *Tapinoma darioi*, *Linepithema humile*, *Lasius neglectus*, and *Lasius grandis*—using 10 replicates per species and season across autumn and spring. Distinct patterns of bait attractiveness were observed among the six studied ant species. *Crematogaster scutellaris*, *P. pallidula*, and *T. darioi* favored protein, whereas invasive *L. humile* and *L. neglectus* preferred carbohydrates. *Lasius grandis* showed no clear preference. Seasonal shifts occurred in some species: *P. pallidula* and *L. neglectus* increased protein recruitment in spring, *L. grandis* favored mixed diets, and *L. humile* shifted further toward carbohydrates. Thus, carbohydrate-rich matrices are indicated for invasive *L. humile* and *L. neglectus*, whereas protein-biased baits suit *C. scutellaris*, *P. pallidula*, and *T. darioi*. Seasonal adjustments, such as increased protein in spring for *P. pallidula*, may enhance bait attractiveness. These findings provide a species-resolved nutritional framework to guide the development of targeted, season-adapted bait formulations for Mediterranean ant pest management.

## Simple Summary

Ants are among the most common urban pests, invading homes, public spaces, and commercial buildings, where they can become a nuisance and interfere with pest management programs. Toxic baits are widely used to control ant infestations, but their success depends on whether ants are attracted to the bait. This study examined the food preferences of six common Mediterranean pest ant species during autumn and spring to identify the bait types most likely to attract each species. We found that some species consistently preferred protein-rich foods, whereas others were more attracted to carbohydrate-rich foods, and several species changed their preferences between seasons. These results show that a single bait formulation may not be equally attractive to all ant pests. Instead, tailoring bait composition to the target species and, when necessary, adjusting formulations according to the season could improve bait acceptance and support control efforts. By providing practical information for the development of more attractive toxic baits, this study contributes to the development of more targeted and potentially effective approaches to urban ant management which may help reduce unnecessary pesticide use.

## 1. Introduction

Ants exhibit considerable diversity in their feeding strategies, ranging from scavenging and predation to the exploitation of plant sap, floral and extrafloral nectar, honeydew, fungi, and seeds [1,2,3,4]. Consequently, nutritional preferences can differ markedly among species. For example, sugar-feeding ants such as *Technomyrmex albipes* and *Linepithema humile* tend to prefer carbohydrate-rich resources [1,5], whereas highly predatory species such as *Brachyponera chinensis* and *Anoplolepis custodiens* preferentially exploit protein-rich food sources [1,6]. Notably, most commercially available bait formulations are optimized for carbohydrate-preferring species and may therefore be less suitable for species with high protein requirements [7]. This mismatch between bait composition and species-specific nutritional preferences represents one of the most underappreciated bottlenecks in the development of effective ant management strategies [1].

At the colony level, ants function as integrated collective units, often described as superorganisms, in which nutritional acquisition and allocation are coordinated among workers, larvae, and queens. The Geometric Framework (GF) for nutrition has provided a powerful conceptual and analytical approach for demonstrating that ant colonies regulate the intake of macronutrients—particularly proteins and carbohydrates—toward colony-specific nutritional targets that support growth, survival, and reproduction [8,9,10,11,12]. When available resources are nutritionally imbalanced, colonies may compensate by over-consuming one nutrient to obtain sufficient amounts of another or by minimizing overall nutritional deviation [8,9,10,11,12]. These trade-offs are particularly relevant because nutritional imbalance can impose costs such as protein toxicity, reduced worker survival, and impaired colony performance [9,10]. Nutritional regulation is also influenced by caste-specific requirements: adult workers rely predominantly on carbohydrates to support metabolism and foraging activity, whereas larvae and queens are major sinks for nitrogen-rich resources required for growth and reproduction [13,14,15]. Larvae additionally process proteinaceous food and redistribute assimilated nutrients within the colony through trophallaxis, contributing to colony-level nutritional regulation [16]. Thus, bait attractiveness is likely to depend not only on the nutritional composition of the bait itself but also on the colony’s current nutritional requirements.

Moreover, colony nutritional requirements are not fixed and may change with environmental conditions and colony phenology. Fluctuations in temperature, photoperiod, and reproductive activity can alter nutritional demands and consequently influence foraging decisions [6,17,18,19,20,21]. Periods of high brood production, typically associated with spring and summer, are often accompanied by increased recruitment to protein-rich resources, whereas cooler periods or late autumn, when brood production may decline, can favor carbohydrate-rich resources supporting worker maintenance and activity [15,22,23,24,25]. Consequently, seasonal variation in nutritional requirements may influence bait attractiveness, meaning that a formulation that is attractive at one time of year may be less attractive at another.

However, comparative information on seasonal variation in nutritional preferences among co-occurring pest ant species remains scarce, particularly in Mediterranean ecosystems [7]. Thus, understanding species-specific nutritional preferences and how these preferences vary over time is an important step toward developing effective ant management strategies. The Mediterranean region presents a particularly urgent management context, hosting a diverse assemblage of ant species that are problematic in domestic environments and crop production systems due to their invasive behavior, pest status, or broader ecological and economic impacts. These include *Linepithema humile*, *Lasius neglectus*, *Tapinoma darioi*, *Crematogaster scutellaris*, *Pheidole pallidula*, and *Lasius grandis* [26,27,28,29,30,31,32,33,34,35]. Despite their management relevance, no comparative study has yet examined their nutritional preferences across seasons, leaving a critical gap in the knowledge base needed to develop targeted, season-appropriate bait formulations for this region. The present study addresses this gap by assessing the macronutrient preferences of these six species across two contrasting seasons—autumn and spring. By addressing this question, this study aims to provide a species-specific, seasonally informed nutritional framework to guide the rational design of targeted bait formulations for ant pest management in urban and periurban areas.

## 2. Materials and Methods

### 2.1. Ant Species and Study Areas

Six ant species occurring in the Mediterranean region and considered problematic either due to pest behavior, invasiveness or broader ecological and economic impacts were selected for this study. The species were chosen to represent three subfamilies (Dolichoderinae, Formicinae, and Myrmicinae), two colony organization types (unicolonial and multicolonial), and two invasion categories (invasive and non-invasive/native), enabling a structured comparative analysis of macronutrient preferences across these biological axes.

Dolichoderinae: The two species representing the subfamily Dolichoderinae were *Linepithema humile* (Mayr, 1868) and *Tapinoma darioi* Seifert, 2017.

*Linepithema humile* is listed among the 100 worst invasive alien species worldwide [36]. Native to South America, it currently invades natural ecosystems, agricultural systems, and urban environments on all continents except Antarctica [30]. In Europe, it is mainly distributed across Mediterranean countries, although it has also been recorded in more continental countries such as Germany, England and the Netherlands, where it appears to be primarily associated with buildings and other indoor environments [29,30]. The species exhibits a unicolonial, polygynous social structure and forms extensive supercolonies [28,37]. The study area used to assess the nutritional preferences of this species was an urban park located along a river in the city of Girona, Spain (Table 1).

*Tapinoma darioi* belongs to the *Tapinoma nigerrimum* complex (Nylander, 1856), which is native to the western and central Mediterranean basin. *Tapinoma darioi* is distributed across northeastern Spain, the Balearic Islands, southern France, Corsica, and northwestern Italy. This species exhibits a unicolonial and polygynous structure and has the capacity to form supercolonies [26]. It has been reported as exotic in the Canary Islands [27], and an introduction has also been documented in the Netherlands [26], indicating its potential as a tramp species. Although it has not yet been formally classified as invasive, these characteristics suggest a considerable invasive risk [26]. In the present study, the sampling area for this species was an urban park located in the village of Quart (Girona), Spain, where the species currently dominates the habitat due to its high competitive ability (Table 1).

Formicinae: The two species representing the subfamily Formicinae were *Lasius grandis* Forel, 1909, and *Lasius neglectus* Van Loon et al., 1990.

*Lasius grandis* belongs to the *Lasius niger* species complex and is widely distributed across the Iberian Peninsula. It is also present in the Balearic Islands, southern France, Corsica, Sardinia, Madeira, and the Azores [38]. The species has also been reported as introduced in the Canary Islands [38]. It is an aphid-tending species that occurs in natural, agricultural and urban environments [38]. In urban areas, it is frequently found in parks and gardens, where it often dominates tree canopies. For this reason, it may become a typical garden pest by promoting aphid outbreaks and causing nuisance to humans. In agricultural environments, it can colonise crop trees and disrupt biological control of sap-feeding pests, leading to higher pest densities and potential crop damage in Mediterranean orchards [33]. This species is multicolonial and monogynous. The study area for *L. grandis* consisted of a wooded area within a public park in Banyoles (Girona), Spain (Table 1).

*Lasius neglectus* is presumed to be native to Asia Minor [31]. First detected in Europe in 1990, it has rapidly spread across Europe and parts of Asia, becoming an important global pest in these regions [31]. The species is polygynous, unicolonial, and capable of forming large supercolonies [31]. It is mainly associated with urban habitats, particularly parks and gardens [31]. However, it is also frequently found inside buildings, where it may occupy electrical conduits and damage electrical outlets, telecommunication systems, and swimming pool motors [32], making it an important domestic pest species. The study population was located along a tree-lined promenade in the city of Sabadell (Barcelona), Spain (Table 1).

Myrmicinae: The two species representing the subfamily Myrmicinae were *Crematogaster scutellaris* (Olivier, 1792) and *Pheidole pallidula* (Nylander, 1849). Both species are characteristic of Mediterranean ecosystems and occur in both natural and urban environments.

*Crematogaster scutellaris* is a primarily arboreal species. Its ability to excavate wood to construct galleries makes it a significant pest in the cork industry, as it perforates cork oak bark, reducing its commercial value [34,35]. It can also affect structural wooden elements in buildings, such as beams and false ceilings, and thus become locally problematic in domestic environments. The species is native to the western Mediterranean region and has been introduced into some northern European countries and the United Kingdom through timber transport [38]. It is a multicolonial and monogynous species. The study area for this species was located at the Montilivi Campus of the University of Girona, where colonies were sampled from several trees near the Faculty of Sciences (Table 1).

*Pheidole pallidula* is a typical Mediterranean species distributed from the Iberian Peninsula to Anatolia [38]. It occurs in natural, agricultural and urban habitats. While *P. pallidula* is an important component of native ant assemblages in Mediterranean habitats [39], it can become problematic in anthropogenic environments. In agricultural systems, it may colonise crop trees and disrupt biological control by attending honeydew-producing pests, potentially increasing pest densities [33]. In urban and domestic settings, it can enter houses in search of food, causing nuisance to householders. The species is polymorphic and multicolonial, typically monogynous, and has an omnivorous diet [38,40]. The study area for this species was located in a semi-urban area near a typical Mediterranean cork oak forest in Santa Cristina d’Aro (Girona), Spain (Table 1).

For each species, the study area was selected based on the presence of a well-established, actively foraging population, identifiable by continuous trail activity and confirmed species identity prior to sampling. These areas were chosen to reflect the typical urban or peri-urban habitat in which each species most commonly occurs as a pest in the Mediterranean region. The study areas’ characteristics are summarized in Table 1.

**Table 1:** Characteristics of the study areas used to assess macronutrient preferences of the six Mediterranean pest ant species studied in this study. Habitat = habitat type; U = urban; PU = peri-urban; Uni = unicolonial; Multi = multicolonial; Inv = invasive; Nat = native. * Nat = native but with invasive potential [40].

Species	Colony Org.	Status	Locality	Habitat	Coordinates	Autumn Sampling	Spring Sampling
Dolichoderinae							
*L. humile*	Uni	Inv	Girona (urban park, riverside)	U	41°59′26.7″ N 2°49′06.1″ E	26 September 2024	19 May 2025
*T. darioi*	Uni	Nat *	Quart, Girona (urban park)	U	41°56′26.6″ N 2°50′23.4″ E	18 September 2024	12 May 2025
Formicinae							
*L. grandis*	Multi	Nat	Banyoles, Girona (wooded park)	U	42°07′38.3″ N 2°45′43.4″ E	1 October 2024	25 May 2025
*L. neglectus*	Uni	Inv	Sabadell, Barcelona (tree-lined promenade)	U	41°33′52.0″ N 2°05′01.9″ E	16 September 2024	20 May 2025
Myrmicinae							
*C. scutellaris*	Multi	Nat	Montilivi Campus, Univ. Girona	U	41°57′46.1″ N 2°49′45.9″ E	29 September 2024	18 May 2025
*P. pallidula*	Multi	Nat	Santa Cristina d’Aro, Girona (peri-urban, cork oak forest edge)	PU	41°50′13.3″ N 3°00′48.0″ E	30 September 2024	21 May 2025

### 2.2. Sampling

The sampling protocol consisted of offering three bait types derived from the artificial ant diet described by Bhatkar and Whitcomb [41]. This gelatin-based diet combines carbohydrates and proteins and has been widely used in ant studies due to its high attractiveness and palatability [42]. Agar was used as a binding agent, with honey serving as the carbohydrate source and egg as the protein source.

The three bait types tested were: (i) the standard Bhatkar diet (mixed macronutrients), (ii) a protein-only diet prepared by removing honey, and (iii) a carbohydrate-only diet prepared by removing egg. All three bait types were offered ad libitum on 5 × 5 cm white plastic plates to prevent depletion effects. Focusing on these two macronutrients allowed us to test dietary preferences because proteins and carbohydrates constitute the primary macronutrients exploited by Mediterranean ants [43].

The three bait types were simultaneously placed at each replicate site, with the three baits within a replicate constituting a single experimental unit for assessing relative recruitment. Their colocation provided a simultaneous three-choice test, allowing workers to choose among the three nutritional alternatives. For each species and season, 10 replicates were conducted. For multicolonial species, each replicate corresponded to a different colony. For unicolonial species, where colonies cannot be reliably distinguished within the same supercolony, replicates were separated by a minimum distance of 3 m to reduce the likelihood of sampling the same foraging population. This spacing followed the approach used in previous bait-choice experiments with *L. humile*, where replicate stations were separated by a minimum distance of 3 m [22]. The baits were placed immediately adjacent to the entrance of an active nest of the target species. No non-target ant species were observed on the baits during the experiments, and no bait stations were displaced, overturned, or otherwise disturbed by wildlife or other external factors. Consequently, no replicates were excluded from the analysis.

Ant recruitment was assessed by a single observer 90 min after bait placement by counting the number of individuals actively feeding at each bait. This observation period was selected to allow sufficient time for bait discovery and subsequent recruitment, while minimizing potential changes in environmental conditions during the assay. Counts were conducted during peak foraging hours (15:00–19:00 h) to minimize variation due to circadian foraging rhythms. Ambient temperature at the time of sampling was recorded at each replicate but was not included as a covariate in the statistical analyses because seasonal variation in temperature was intrinsically associated with the season factor and could not be separated from it given the study design (mean temperature spring: 28.3 ± 2.8 °C; mean temperature autumn: 25.2 ± 6.3 °C). Recruitment counts at bait stations are a widely used proxy for nutritional preference in field-based cafeteria assays, as they integrate the colony’s collective foraging decision and are strongly correlated with bait consumption in ants under natural conditions [1].

Sampling was conducted during autumn (late September-early October 2024) and spring (mid to late May 2025), corresponding to the two key phases of the annual colony cycle in Mediterranean ant species: the pre-hibernation accumulation phase and the reproductive onset phase, respectively.

### 2.3. Statistical Analysis

We analyzed species-specific resource preferences using a multinomial logistic regression model fitted with the *multinom* function in the *nnet* package in R. The response variable was the diet type (categorical: mixed, protein or carbohydrate), and species was included as a predictor. For each replicate, the numbers of workers recorded at the three bait types were used to estimate the relative distribution of worker recruitment among diet types. The total number of workers recorded across the three baits in that replicate was included as a model weight. This weighting accounted for the substantial variation in the number of workers contributing to the observed resource preferences among species and replicates. Thus, replicates based on larger numbers of workers contributed more information to the estimated probabilities than replicates based on only a few workers, without altering the relative distribution of workers among diet types within each replicate.

This model was used to characterize interspecific variation in bait preference across the study period, using observations from both autumn and spring. The resulting model estimates were used to obtain predicted probabilities of selecting each bait type for each species. To evaluate seasonal changes in nutritional preferences, we fitted a second multinomial model including species, season (autumn vs. spring), and their interaction (species × season) as predictors. Predicted probabilities from this second model were obtained for each species and season, allowing seasonal variation in bait preference to be assessed.

Estimated marginal means (*emmeans*) were calculated to derive the predicted probability of selecting each bait, with 95% confidence intervals. Post hoc pairwise comparisons between bait types within each species were performed using Tukey-adjusted contrasts to identify significant preferences.

All statistical analyses were conducted in R [44] (version 4.4.3) and graphical representations were generated using ggplot2.

## 3. Results

### 3.1. Species-Specific Nutritional Preferences

Nutritional preferences differed markedly among the six ant species studied when considering the full sampling period (Figure S1). Three species showed a significant preference for protein-based baits: *C. scutellaris*, *P. pallidula*, and *T. darioi* all recruited preferentially to protein (*C. scutellaris*: prob = 0.583, SE = 0.026, CI = 0.525–0.641; *P. pallidula*: prob = 0.554, SE = 0.034, CI = 0.479–0.629; *T. darioi*: prob = 0.603, SE = 0.023, CI = 0.551–0.655). Post hoc contrasts confirmed significant differences in protein preference compared with the other diets (i.e., carbohydrate and mixed) in all three species (all contrasts: *p* < 0.05) (Figure S1).

Two species recruited preferentially carbohydrate-based baits: *L. humile* and *L. neglectus* (*L. humile*: prob = 0.446, SE = 0.010, CI = 0.423–0.468; *L. neglectus*: prob = 0.468, SE = 0.018, CI = 0.427–0.509). Post hoc contrasts confirmed these differences for *L. humile* (carbohydrate vs. protein and carbohydrate vs. mixed diet: *p* < 0.001), whereas for *L. neglectus*, only the preference for carbohydrates over protein was significant (t = −13.571; *p* < 0.0001), with no significant difference between carbohydrate and mixed diet (t = −1.635; *p* = 0.269).

*Lasius grandis* showed no clear preference among diets (mixed: prob = 0.438 ± 0.045, CI 0.339–0.536; protein: prob = 0.248 ± 0.039, CI 0.162–0.333; carbohydrate: prob = 0.314 ± 0.042, CI 0.222–0.406), a pattern further supported by post hoc contrasts, which revealed no significant differences between them (all contrasts: *p* > 0.05) (Figure S1).

### 3.2. Seasonal Shifts in Nutritional Preferences

**Figure 1:**
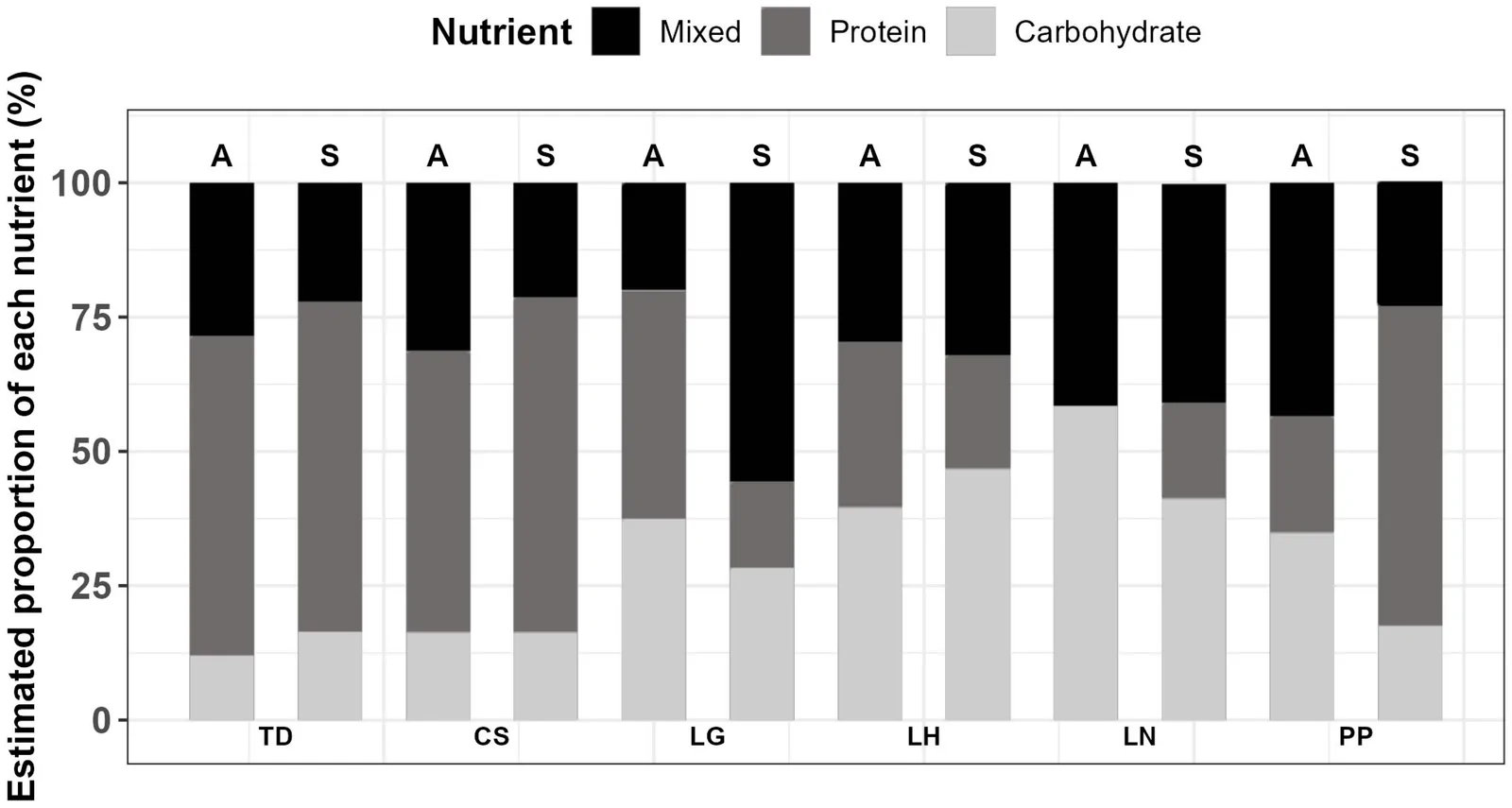
Seasonal variation in the estimated proportions of each bait type (mixed, protein, and carbohydrate) for each ant species, based on the second multinomial model including species, season, and their interaction. Fills represent the model-estimated probability of selecting each bait type for each species and season. Values are expressed as percentages (%) and shown as stacked bars. Autumn (A); spring (S). LH: *Linepithema humile*; TD: *Tapinoma darioi*; LN: *Lasius neglectus*; LG: *Lasius grandis*; CS: *Crematogaster scutellaris*; PP: *Pheidole pallidula*.

Seasonal patterns differed among species. *Crematogaster scutellaris* and *T. darioi* maintained similar patterns in both autumn and spring (Figure 1). For *C. scutellaris* and *T. darioi*, protein remained the preferred nutrient across seasons (Table 2). *Lasius neglectus* retained the same preference ranking across seasons, consistently avoiding protein-based baits; however, recruitment to protein increased significantly in spring, from near zero in autumn to 0.179 ± 0.017, together with a significant decrease in recruitment to carbohydrate baits (Figure 1, Table 2).

The remaining three species showed season-dependent shifts in nutritional preferences. *Linepithema humile* exhibited an increased preference for carbohydrates in spring relative to autumn, with a corresponding reduction in protein recruitment (Table 2). *Pheidole pallidula* displayed a pronounced increase in protein preference during spring (Table 2), consistent with increased colony demand for nitrogen-rich resources during the reproductive season. *Lasius grandis* showed a higher preference for a mixed diet in spring (Table 2).

## 4. Discussion

The results of this study demonstrate that macronutrient preferences are strongly species-specific among the studied species of Mediterranean ant pests, with marked differences observed across the six species studied, even among ecologically similar taxa. This interspecific variation has direct consequences for bait-based management as bait attractiveness is an essential component of bait efficacy: if a bait is not sufficiently attractive relative to naturally available food sources, its potential for successful toxicant delivery and colony control is inherently limited. These findings therefore highlight the potential value of tailoring bait nutritional profiles to the target species to enhance bait attractiveness and potentially improve bait efficacy under field conditions. A particularly striking finding is the divergence in macronutrient preferences among honeydew-tending species. Four of the six species studied—*Lasius grandis*, *L. neglectus*, *T. darioi*, and *C. scutellaris*—are typically considered honeydew-tending ants [26,38,45], for which a predominant carbohydrate preference might be expected. However, their preferences diverged substantially. *Lasius neglectus* showed a preference for carbohydrate-rich baits and comparatively low recruitment to protein-based baits, consistent with its reliance on honeydew as a primary energy source [46] and with its classification as a sugar-preferring generalist by Arnan et al. [43]. *Lasius grandis*, by comparison, displayed a more balanced profile, with no clear overall preference across the study period and substantial recruitment to protein-based baits in autumn. In contrast, *C. scutellaris* and *T. darioi* exhibited a strong and consistent preference for protein in both seasons, a pattern consistent with Arnan et al. [43], who classified *C. scutellaris* as a generalist with a marked preference for protein over sugar, but unexpected given the honeydew-tending habits of both species. This suggests that honeydew-tending behavior does not necessarily translate into a colony-level carbohydrate preference, and that other factors—including foraging ecology, colony size, or the local availability of alternative protein-rich food sources—may drive interspecific divergence even among functionally similar species.

The divergence between *L. neglectus* and *L. grandis* is particularly informative in this regard. *Lasius neglectus* avoided protein-based baits almost entirely in autumn and, despite the significant increase in spring, kept protein as its least preferred resource; its autumn recruitment to protein was far below that of *L. grandis*. This is consistent with field observations showing that *L. grandis* captures a higher proportion of insects than *L. neglectus*—9.91% vs. 3.48% of workers carrying prey, respectively [46]—suggesting that the invasive *L. neglectus* has a more restricted dietary niche than its native congener, with a stronger dependence on liquid carbohydrate sources. From a management perspective, these results suggest that carbohydrate-based bait formulations may be more attractive to *L. neglectus*, whereas mixed or seasonally adjusted matrices may better suit *L. grandis*, while protein-based matrices may elicit greater recruitment in *C. scutellaris* and *T. darioi*— although the extent to which recruitment differences translate into colony-level toxicant uptake and control efficacy would need to be validated in field trials.

Beyond the variation among honeydew-tending species, the carbohydrate bias described above for *L. neglectus* was mirrored by the other invasive species studied here, *L. humile*, despite their differing overall dietary profiles. The strong carbohydrate preference of *L. humile* is consistent with previous studies showing that this species predominantly exploits liquid, sugar-rich resources, particularly hemipteran honeydew [14,15], and the present results extend this pattern to urban habitats, suggesting that the carbohydrate-oriented foraging strategy of *L. humile* is robust across ecological contexts. This convergence between the two invaders aligns with numerous studies reporting carbohydrate preference among successful invasive ants, including *Crematogaster peringueyi* [1,47], *Pheidole megacephala* [48], *Solenopsis invicta* [19], *Anoplolepis gracilipes* [49], and *Technomyrmex albipes* [5]. This pattern is not universal—specialized predatory invasive species such as *Brachyponera chinensis* prioritize protein-rich prey [6]—but the convergence observed here, together with the broader literature, suggests that carbohydrate reliance may represent a nutritional strategy particularly associated with tramp and supercolonial invasive ants that exploit liquid sugar-rich resources in human-modified environments.

A possible explanation for this widespread pattern is that carbohydrate availability can directly influence behavioral dominance in ants. Increased carbohydrate intake may enhance activity, foraging intensity, and aggressiveness in ant species [50,51,52] (but see [53,54] for contrasting responses). For example, *A. gracilipes* carbohydrate consumption supports a high-tempo foraging strategy associated with ecosystem dominance [52], while *L. humile* carbohydrate scarcity reduces both activity and aggressive interference [51]. Similarly, *S. invicta* actively extracts carbohydrates from mixed resources to meet high energetic demands [9]. Under this hypothesis, a carbohydrate-oriented nutritional strategy may sustain the energetically demanding behaviors—sustained foraging, mass recruitment, and aggressive competitor displacement—that characterize ecologically dominant invasive species. While our study does not directly test this mechanism, the convergent carbohydrate preference observed across multiple invasive taxa is consistent with this interpretation and warrants further experimental investigation. From an applied standpoint, the consistent carbohydrate recruitment preference of both invasive species provides empirical support for the use of sugar-based bait matrices as the primary formulation strategy for *L. humile* and *L. neglectus* management in Mediterranean urban environments, pending field validation of colony-level efficacy.

In addition to interspecific variation, seasonal shifts in nutritional preferences were evident in four of the six species studied, though the direction and magnitude of these shifts were not uniform, reflecting the diversity of colony phenologies among Mediterranean ant pests. Autumn typically represents a period of resource accumulation in species that overwinter, whereas spring is characterized by intense reproductive activity, with queens laying eggs and large numbers of larvae present in the nest—conditions that are generally expected to increase colony demand for protein [6,15,17,22,24,25]. This expected spring increase in protein preference was observed in *P. pallidula*, which showed a pronounced shift toward protein recruitment in spring, consistent with the elevated nitrogen demands associated with brood development and queen oviposition. *Lasius neglectus*, while still prioritizing carbohydrates in spring, also showed a significant increase in recruitment to protein-based baits relative to autumn—a partial shift that may reflect a constrained but real colony-level demand for protein during the reproductive period. *Crematogaster scutellaris* and *T. darioi* maintained a strong protein preference across both seasons, suggesting that their high colony-level protein demand is a year-round nutritional characteristic rather than a seasonally driven response.

However, this seasonal trend was not observed in *L. humile*. Rather than increasing protein recruitment in spring, carbohydrate recruitment increased, contrary to previous reports of higher protein intake during this season [22]. This pattern may be related to the timing of our spring sampling, conducted in May, which coincides with a specific phase of the *L. humile* annual cycle characterized by massive queen elimination and declining queen density within nests [55,56]. At the same time, pupae become abundant while larval numbers begin to decline [57], suggesting a shift in colony-level protein demand relative to earlier in spring. The increased carbohydrate preference observed may therefore reflect the colony’s shift toward worker maintenance and sustained foraging activity at a moment when reproductive demands have temporarily subsided. Notably, *L. humile* colonies are known to increase prey intake earlier in spring [15], consistent with the higher abundance of larvae and elevated egg-laying rates observed in nests at that time [13,57,58], suggesting that the timing of protein demand in this species peaks before May. Further studies spanning the full spring period would be valuable to test this hypothesis and determine whether the observed shift in bait preference is associated with seasonal changes in colony reproductive activity.

The preference of *L. grandis* for a mixed diet in spring, rather than a clear shift toward protein, may similarly reflect a nutritional buffering strategy. Choosing a mixed nutrient source may allow the colony to function as a “collective gut” [16], enabling workers to selectively extract carbohydrates for their own energetic needs while retaining protein for larval development [9]. Given that excessive protein intake without sufficient carbohydrates reduces worker lifespan [16] and that the elevated foraging activity of spring imposes substantial metabolic demands [19], a mixed-diet preference may represent an adaptive strategy that balances the competing nutritional needs of workers and brood simultaneously. Collectively, these results indicate that seasonal bait adjustments cannot be applied uniformly across species. Incorporating protein into spring bait formulations may enhance recruitment against *P. pallidula* but is unlikely to improve attractiveness for species that remain carbohydrate-biased in spring such as *L. humile* and *L. neglectus*. These findings highlight the need for species-specific seasonal bait strategies in Mediterranean ant pest management programs.

The present study provides a first comparative assessment of bait attractiveness in six Mediterranean pest ant species using honey and egg as carbohydrate and protein sources, respectively, following the widely used Bhatkar diet [41]. Previous research has reported variability in ant preferences for different types of carbohydrates [5,18,43,48,59] and proteins [6,20,43] as well as differences in responses to solid versus liquid bait matrices [1,20]. Although each species was represented by a single population in the present study, the observed patterns provide a useful basis for comparative assessment of bait attractiveness among Mediterranean pest ants. Further studies including multiple populations and a wider range of nutrient sources and bait types would help determine the consistency of these patterns across populations and allow finer characterization of species-specific nutritional profiles, ultimately contributing to the development of more effective and targeted bait formulations for ant pest management.

## 5. Conclusions

This study shows that bait attractiveness differed markedly among the six Mediterranean pest ant species examined and changed between seasons in several of them. Because attractiveness does not necessarily equate to consumption or control efficacy, additional field trials using toxic formulations are required before management recommendations can be generalised. Nevertheless, these findings support moving beyond generic formulations toward species- and season-informed nutritional strategies. Aligning bait composition with the macronutrient preferences of the target species—protein-biased for *C. scutellaris*, *P. pallidula* and *T. darioi*, carbohydrate-based for *L. humile* and *L. neglectus*; and mixed matrices for *L. grandis*—and adjusting formulations seasonally where relevant, provides an evidence-based starting point for developing more attractive bait formulations and may contribute to more consistent control of ant pests in urban and periurban environments.

## Figures and Tables

**Table 2:** Estimated probability of worker recruitment to each bait type (Prob ± SE [95% CI]) for the six ant species studied across Autumn and Spring. Asterisks indicate significant seasonal differences between spring and autumn (Holm-adjusted pairwise comparisons): * *p* < 0.05, ** *p* < 0.01, *** *p* < 0.001. † Estimate = 1.00 × 10^−7^ ± 2.00 × 10^−8^; 95% CI = 1.00 × 10^−7^–2.00 × 10^−7^.

Species	Diet Type	Autumn (Prob ± SE [CI])	Spring (Prob ± SE [CI])
*C. scutellaris*	Mixed	0.312 ± 0.039 [0.232–0.393]	0.213 ± 0.029 [0.153–0.272]
*C. scutellaris*	Protein	0.525 ± 0.042 [0.438–0.612]	0.624 ± 0.034 [0.553–0.694]
*C. scutellaris*	Carbohydrate	0.163 ± 0.031 [0.099–0.227]	0.163 ± 0.026 [0.110–0.217]
*T. darioi*	Mixed	0.285 ± 0.029 [0.224–0.345]	0.220 ± 0.031 [0.156–0.283]
*T. darioi*	Protein	0.594 ± 0.032 [0.529–0.660]	0.615 ± 0.036 [0.541–0.690]
*T. darioi*	Carbohydrate	0.121 ± 0.021 [0.078–0.165]	0.165 ± 0.028 [0.108–0.222]
*L. neglectus*	Mixed	0.416 ± 0.032 [0.350–0.483]	0.408 ± 0.022 [0.362–0.454]
*L. neglectus*	Protein ***	<0.001 †	0.179 ± 0.017 [0.143–0.215]
*L. neglectus*	Carbohydrate **	0.584 ± 0.032 [0.517–0.650]	0.412 ± 0.022 [0.366–0.459]
*L. humile*	Mixed	0.294 ± 0.017 [0.259–0.330]	0.321 ± 0.011 [0.297–0.344]
*L. humile*	Protein **	0.310 ± 0.018 [0.274–0.346]	0.212 ± 0.010 [0.192–0.233]
*L. humile*	Carbohydrate *	0.396 ± 0.019 [0.358–0.434]	0.467 ± 0.012 [0.442–0.492]
*P. pallidula*	Mixed	0.435 ± 0.103 [0.221–0.648]	0.229 ± 0.031 [0.166–0.292]
*P. pallidula*	Protein **	0.217 ± 0.086 [0.040–0.395]	0.596 ± 0.036 [0.522–0.670]
*P. pallidula*	Carbohydrate	0.348 ± 0.099 [0.143–0.553]	0.176 ± 0.028 [0.118–0.233]
*L. grandis*	Mixed **	0.200 ± 0.063 [0.070–0.331]	0.556 ± 0.055 [0.442–0.670]
*L. grandis*	Protein	0.425 ± 0.078 [0.264–0.586]	0.160 ± 0.041 [0.076–0.245]
*L. grandis*	Carbohydrate	0.375 ± 0.077 [0.217–0.533]	0.284 ± 0.050 [0.181–0.387]

## Data Availability

The dataset supporting the findings of this study is publicly available in Zenodo at https://doi.org/10.5281/zenodo.21132290.

## Supplementary Materials

The following supporting information can be downloaded at https://www.mdpi.com/article/10.3390/insects17090945/s1. Figure S1: Estimated proportions of each nutrient (mixed, protein, carbohydrate) for each ant species, based on the multinomial model using observations collected across both autumn and spring. LH: *Linepithema humile*; TD: *Tapinoma darioi*; LN: *Lasius neglectus*; LG: *Lasius grandis*; CS: *Crematogaster scutellaris*; PP: *Pheidole pallidula*.
